# An essential regulatory function of the DnaK chaperone dictates the decision between proliferation and maintenance in *Caulobacter crescentus*

**DOI:** 10.1371/journal.pgen.1007148

**Published:** 2017-12-27

**Authors:** Frederic D. Schramm, Kristina Heinrich, Marietta Thüring, Jörg Bernhardt, Kristina Jonas

**Affiliations:** 1 Science for Life Laboratory, Department of Molecular Biosciences, The Wenner-Gren Institute, Stockholm University, Stockholm, Sweden; 2 LOEWE Center for Synthetic Microbiology (SYNMIKRO), Philipps University Marburg, Marburg, Germany; 3 Institute of Microbiology, Ernst-Moritz-Arndt University Greifswald, Greifswald, Germany; University of Geneva Medical School, SWITZERLAND

## Abstract

Hsp70 chaperones are well known for their important functions in maintaining protein homeostasis during thermal stress conditions. In many bacteria the Hsp70 homolog DnaK is also required for growth in the absence of stress. The molecular reasons underlying Hsp70 essentiality remain in most cases unclear. Here, we demonstrate that DnaK is essential in the α-proteobacterium *Caulobacter crescentus* due to its regulatory function in gene expression. Using a suppressor screen we identified mutations that allow growth in the absence of DnaK. All mutations reduced the activity of the heat shock sigma factor σ^32^, demonstrating that the DnaK-dependent inactivation of σ^32^ is a growth requirement. While most mutations occurred in the *rpoH* gene encoding σ^32^, we also identified mutations affecting σ^32^ activity or stability *in trans*, providing important new insight into the regulatory mechanisms controlling σ^32^ activity. Most notably, we describe a mutation in the ATP dependent protease HslUV that induces rapid degradation of σ^32^, and a mutation leading to increased levels of the house keeping σ^70^ that outcompete σ^32^ for binding to the RNA polymerase. We demonstrate that σ^32^ inhibits growth and that its unrestrained activity leads to an extensive reprogramming of global gene expression, resulting in upregulation of repair and maintenance functions and downregulation of the growth-promoting functions of protein translation, DNA replication and certain metabolic processes. While this re-allocation from proliferative to maintenance functions could provide an advantage during heat stress, it leads to growth defects under favorable conditions. We conclude that *Caulobacter* has co-opted the DnaK chaperone system as an essential regulator of gene expression under conditions when its folding activity is dispensable.

## Author summary

Molecular chaperones of the Hsp70 family belong to the most conserved cellular machineries throughout the tree of life. These proteins play key roles in maintaining protein homeostasis, especially under heat stress conditions. In diverse bacteria the Hsp70 homolog DnaK is essential for growth even in the absence of stress. However, the molecular mechanisms underlying the essential nature of DnaK have in most cases not been studied. We found in the α-proteobacterium *Caulobacter crescentus* that the function of DnaK as a folding catalyst is dispensable in the absence of stress. Instead, its sole essential function under such conditions is to inhibit the activity of the heat shock sigma factor σ^32^. Our findings highlight that some bacteria have co-opted chaperones as essential regulators of gene expression under conditions when their folding activity is not required. Furthermore, our work illustrates that essential genes can perform different essential functions in discrete growth conditions.

## Introduction

Hsp70 proteins play a key role in thermal stress adaptation by assisting the folding and refolding of client proteins, thereby preventing and reversing the accumulation of toxic protein aggregates [1]. In addition to this chaperoning activity these proteins can play critical roles in regulating gene expression in response to stress [2]. In *Escherichia coli* and other proteobacteria, the bacterial Hsp70 protein DnaK inhibits the heat shock sigma factor σ^32^ by binding to and targeting it for degradation by the membrane-bound protease FtsH until increasing demand for its folding function titrates the chaperone away to misfolded proteins [3–5]. Liberation of σ^32^ from DnaK in response to unfolded protein stress allows the sigma factor to associate with the RNA polymerase (RNAP) and induce expression of molecular chaperones and cellular proteases, among other physiological changes. These adaptive processes are collectively referred to as the heat shock response. Importantly, σ^32^ also induces the expression of the *dnaK* gene itself. This σ^32^-dependent upregulation of DnaK levels leads to re-sequestration of σ^32^ and a shut-down of the heat shock response through an autoregulatory loop [6–8].

DnaK function has been extensively studied in *E*. *coli*. Deletion of *dnaK* in this organism leads to cell death under heat shock conditions [9]. At intermediate and low temperatures Δ*dnaK E*. *coli* cells are viable but show growth defects [9–11]. That DnaK is dispensable under these conditions has been explained by the high degree of functional redundancy of the chaperone network and the finding that few DnaK clients are essential proteins [9,12–15]. In contrast to the situation in *E*. *coli*, DnaK is essential for growth not only at elevated temperature but also under optimal conditions in other bacterial species [16–20]. In *Mycobacterium smegmatis*, it was shown that DnaK has a non-redundant role in the folding of essential proteins and in generally maintaining protein homeostasis at both high and low temperatures and is therefore required even in the absence of stress [19]. The reasons for DnaK essentiality remain unclear in other bacteria.

Like *M*. *smegmatis*, the α-proteobacterium *Caulobacter crescentus*, which is a model organism for studying bacterial cell cycle regulation, strictly depends on DnaK at all temperature conditions [20,21]. Recent work in this organism identified a previously suggested [22] role for DnaK in controlling DNA replication [23]. It was demonstrated that depletion of DnaK induces degradation of the replication initiator DnaA through upregulation of the protease Lon as part of the heat shock response. Consequently, DnaA is cleared from DnaK-depleted cells and DNA replication initiation inhibited.

Here, we show that in *C*. *crescentus* DnaK not only impacts DNA replication but has an additional important role in maintaining cell growth under favorable conditions. Unexpectedly, depletion of DnaK does not lead to major protein aggregation in *C*. *crescentus* under non-stress conditions. By isolating mutations that permit growth of DnaK-depleted cells we demonstrate that in the absence of stress the sole essential function of DnaK is to inhibit σ^32^. We further find that efficient repression of σ^32^-dependent transcription is a growth requirement and that σ^32^ activation under conditions when DnaK is not available reprograms gene expression on a global scale leading to a re-organization of cellular activities from proliferative to cytoprotective modes.

## Results

### DnaKJ is required for maintaining growth under non-stress conditions independently of its impact on DNA replication

**Fig 1:**
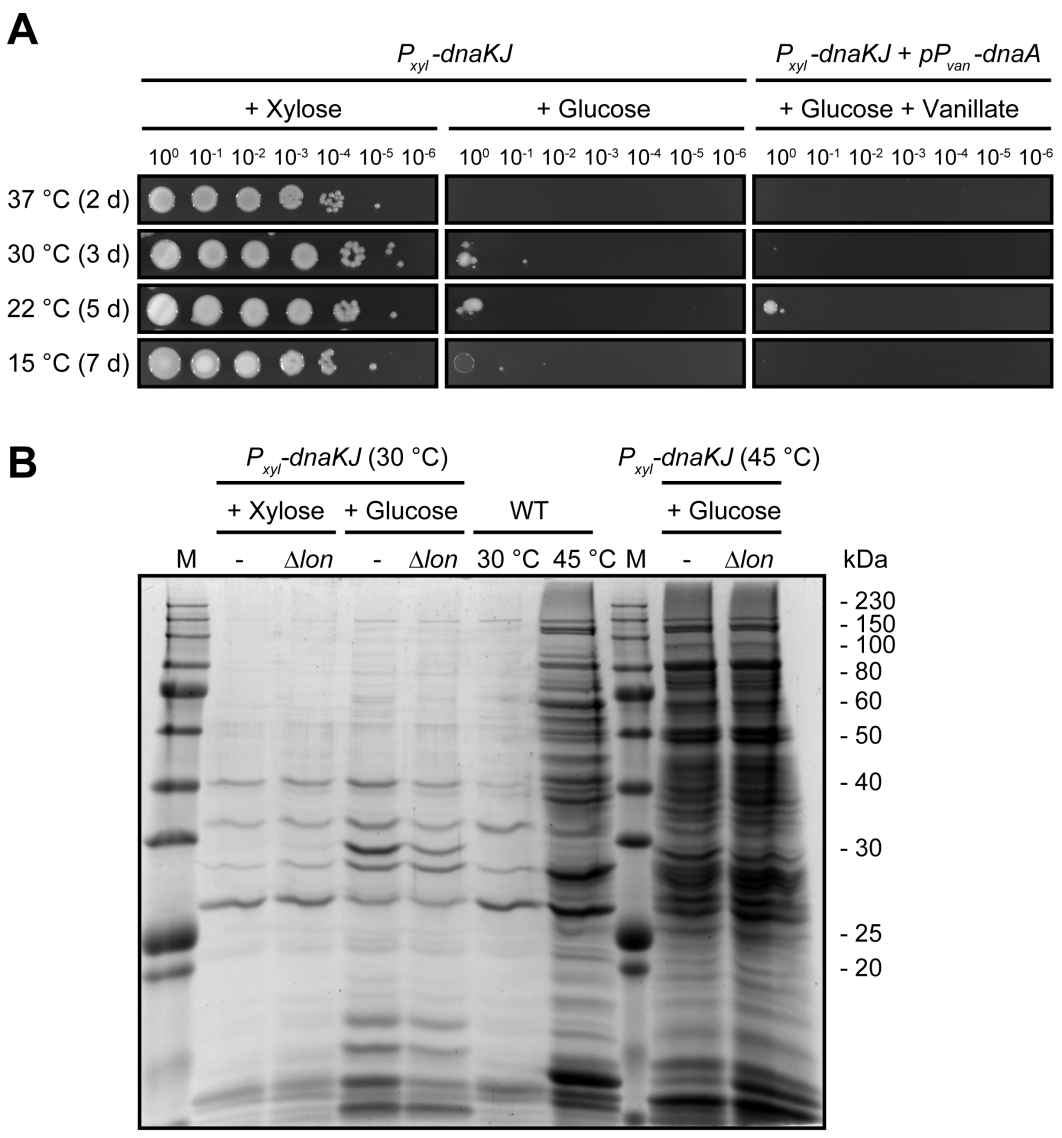
DnaKJ is required for growth under optimal conditions independently of DnaA and its function as a general folding catalyst. (A) Colony formation of a DnaKJ depletion strain in the absence or presence of vanillate-dependent *dnaA* overexpression at different growth temperatures on PYE agar plates. Depleting (+ Glucose) and non-depleting (+ Xylose) conditions are shown. See S1 Fig for additional colony formation assays. (B) Aggregation assay showing the insoluble detergent-resistant protein fractions of the wild type and the DnaKJ depletion strain. DnaKJ was depleted for 24 hours in an otherwise wild type or Δ*lon* background before aggregated proteins were isolated. Cells were either grown constantly at 30°C or incubated for 2 hours at 45°C prior to sampling.

Depletion of the chaperone DnaK with its co-chaperone DnaJ (hereafter DnaKJ) results in growth arrest of *C*. *crescentus* and an inability to form colonies at all temperatures tested (Fig 1A). This phenotype is not growth medium-specific as no differences were observed between incubation on complex and minimal media (S1 Fig). The phenotype of cells depleted either for the main chaperone DnaK alone or its co-chaperone DnaJ mimicked the phenotype of the double depletion demonstrating that both DnaK and DnaJ are essential under the conditions tested (S1 Fig). Previous work demonstrated that DnaKJ depletion in *C*. *crescentus* leads to a strong reduction in abundance of the replication initiator DnaA culminating in a G1-arrest [23] which we hypothesized could explain the essentiality of DnaK both at optimal and lower temperatures. However, restoration of DNA replication by ectopically overproducing DnaA does not restore colony formation in DnaKJ-depleted cells (Fig 1A). This suggests that DnaKJ depletion impairs growth independently of its effect on DnaA.

### DnaKJ depletion at optimal temperatures does not induce a proteostasis collapse

To test if the observed DNA-replication-independent growth defect in cells lacking DnaKJ was due to a collapse of general protein homeostasis (proteostasis), we analyzed global protein aggregation by isolating the insoluble detergent-resistant fraction from cellular lysates (Fig 1B). At 30°C, the standard and optimal growth temperature of *C*. *crescentus*, DnaKJ depletion did not induce strong overall protein aggregation compared to exponentially growing non-depleted cells, both in the presence and in the absence of the protease Lon, the primary protease degrading misfolded proteins in *E*. *coli* [24]. Instead, only a small subset of protein bands appeared or increased in abundance in the aggregate fraction of cells depleted of DnaKJ at 30°C, which could correspond to a limited amount of aggregating DnaK substrates and interacting heat shock proteins that are known to be strongly upregulated after loss of DnaKJ [6,25,26]. Exposing DnaKJ depleted cells to 45°C for two hours caused clearly stronger protein aggregation compared to the wild type, demonstrating the importance of DnaK's chaperone function at high temperature. Together, these data show that DnaKJ depletion under non-stress conditions does not induce drastic protein aggregation, indicating that a proteostasis collapse is not the reason for the observed growth defect in DnaKJ-depleted cells.

### Mutations in genes encoding components of the transcriptional machinery and HslUV bypass the lethality of DnaKJ depletion

**Fig 2:**
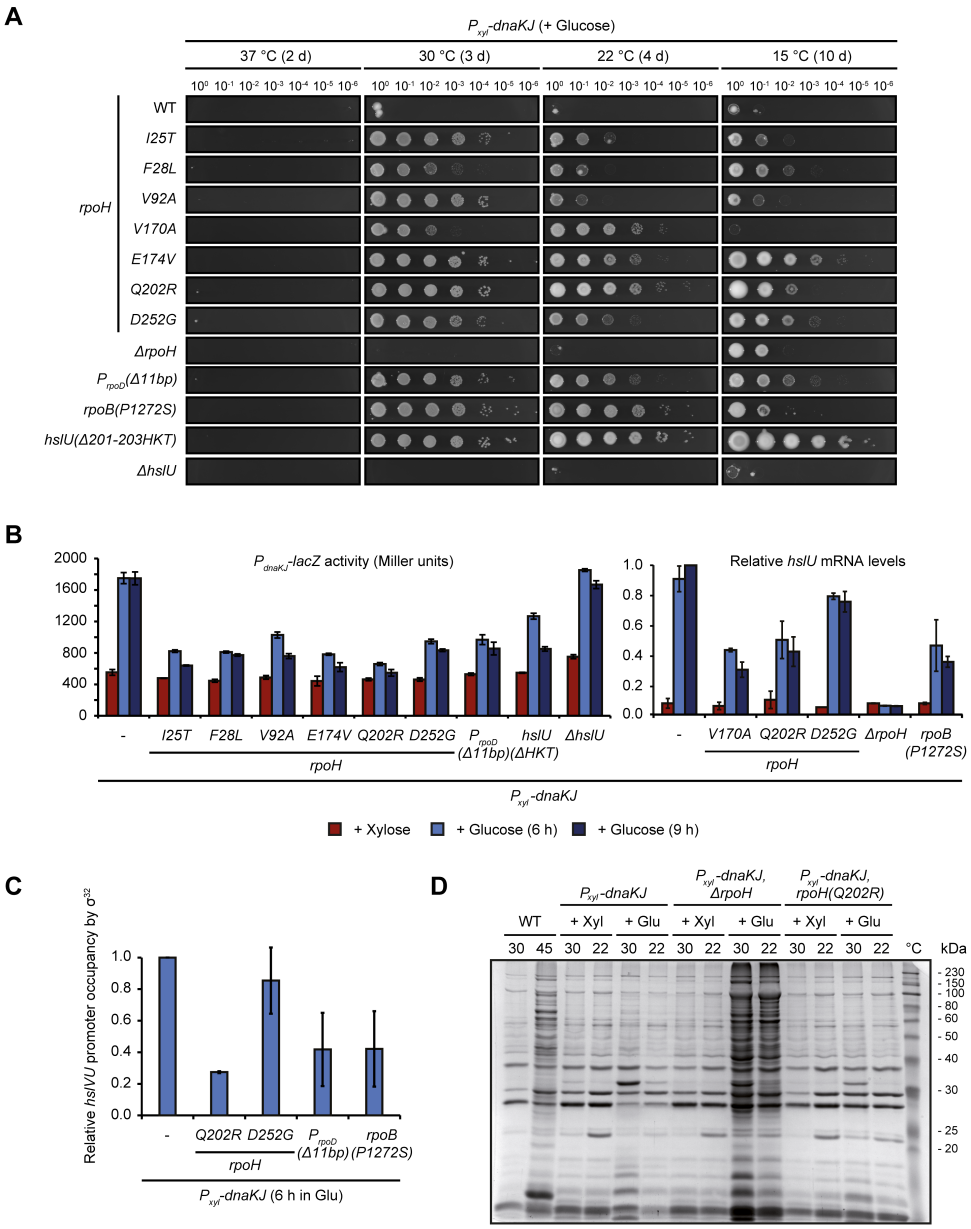
Mutations in *rpoH*, *rpoB*, *hslU* and the promoter of *rpoD* suppress the lethality of DnaKJ depletion. (A) Spot assays comparing colony formation of the parental DnaKJ depletion strain and its derivative strains harboring the re-introduced suppressor mutations as well as a *ΔrpoH* and a *ΔhslU* mutation, respectively, after incubation at 37, 30, 22 and 15°C under depleting conditions for the indicated time. See S2 Fig for illustrations showing the location of the suppressor mutations on protein domain structures and S3 Fig for spot colony formation assays under non-depleting conditions. (B) LacZ-assays monitoring σ^32^-dependent transcription from the *dnaKJ* promotor region and qRT-PCR monitoring *hslU* mRNA levels in the presence of DnaKJ and following 6 or 9 hours of depletion in different genetic backgrounds. qRT-PCR was done instead of LacZ-assays because of difficulties to transform the DnaKJ depletion strains containing *rpoH(V170A)*, *rpoB(P1272S)* and *ΔrpoH* with pDel1, the strains containing *rpoH(Q202R)* and *rpoH(D252G)* are shown for comparison. Quantifications are presented as the means of independent duplicates. Error bars represent standard deviations. (C) qChIP data presenting *hslVU* promoter occupancy by σ^32^ in different genetic backgrounds after depleting DnaKJ for six hours. Quantifications are based on two independent replicates. Error bars represent standard deviations. (D) Coomassie stained SDS-PAGE showing the insoluble detergent-resistant protein fractions of the wild type grown at 30°C or shifted to 45°C for 1 h as well as the DnaKJ depletion strain and its genetic derivatives under depleting (+ Glu) and non-depleting (+ Xyl) conditions at 30°C and 22°C. Cells were depleted for 24 h when grown at 30°C and for 48 h when grown at 22°C.

We searched for mutations that can restore growth in the absence of DnaKJ using a suppressor screen (see Material and Methods for a detailed description of the screening procedure). Mutations identified in the screen mapped to genes encoding different components of the transcriptional apparatus as well as a subunit of an ATP-dependent protease. We found five mutations in *rpoH*, the gene encoding σ^32^, one mutation in *rpoB*, which codes for the β-subunit of the RNAP, one mutation upstream of *rpoD* encoding the house keeping sigma subunit σ^70^ of the RNAP and one mutation in the *hslU* gene, which encodes the ATPase subunit of the protease HslUV (S2 Fig). The mutations in *rpoH* and *rpoB* were missense mutations leading to amino acid replacements. The mutation upstream of *rpoD* was an 11 bp-deletion, affecting the -10 region of one of the promoters. The mutation in *hslU* was a 9 bp-deletion removing three amino acids (Δ201-203HKT) in the substrate recognition domain of HslU (S2 Fig). To confirm that the suppression of growth defects observed in the clones isolated in our suppressor screen are linked to these mutations, we re-introduced them into the parental DnaKJ-depletion strain (SG400) harboring no other genetic modifications or plasmids. Re-introduction of each of these mutations restored growth in the absence of DnaKJ (Fig 2A). Based on this result, we used the strains with the re-introduced suppressor mutations in all following experiments. We assessed colony formation of these strains at different temperatures and observed that the degree of suppression was temperature-dependent and varied between the different mutants. Most notably, the *rpoH(V170A)* allele suppressed loss of DnaKJ well at 22°C and 30°C, but failed to restore colony formation at 15°C (Fig 2A). Furthermore, under non-depleting conditions this mutation prevented colony formation at 37°C, at which the parental strain and all other suppressor mutants readily form colonies (S3 Fig). All together, we isolated several mutations capable of restoring growth at the physiological temperature of 30°C in the absence of DnaKJ.

### Partial or complete loss of σ^32^ function restores growth of DnaKJ-depleted cells

Previously identified loss-of-function point mutations in *rpoH* have been shown to weaken heat shock gene induction [23,27]. Consistent with these data, all suppressor mutations we identified that mapped to *rpoH* led to reduced σ^32^-dependent gene expression following DnaKJ depletion as monitored by LacZ-assays using a *P*_*dnaKJ*_*-lacZ* reporter plasmid [28] or, in strains that exhibited difficulties maintaining the plasmid, by quantifying mRNA levels of the σ^32^-controlled *hslU* gene (Fig 2B). Western blot analysis after nine hours of DnaKJ-depletion revealed no significant differences between σ^32^-levels in the wild type background and strains harboring the different *rpoH* alleles (S4 Fig), suggesting these mutations reduce transcriptional activity rather than protein stability. In accordance with this, quantitative chromatin immunoprecipitation (qChIP) experiments with two of the σ^32^ mutants, σ^32^(Q202R) and σ^32^(D252G), showed a decrease in σ^32^ occupancy of the *hslVU* operon promoter after six hours of DnaKJ-depletion (Fig 2C).

We wondered if complete absence of σ^32^ could also suppress the lack of DnaKJ, and deleted the *rpoH* gene from the DnaKJ depletion strain. Under non-depleting conditions this strain formed colonies at intermediate (30°C and 22°C) and low temperatures (15°C), but not at 37°C, similar to the strain harboring the V170A mutation in *rpoH* (S3 Fig). Spot assays showed that at 15°C the *rpoH* deletion strain indeed suppressed the lethal effect of the DnaKJ depletion (Fig 2A). However, unlike the *rpoH* suppressor mutations, deletion of *rpoH* did not allow growth at 30°C or 22°C, even though the block in DNA replication after loss of DnaKJ was alleviated (S5 Fig). Given the complete absence of heat shock response induction in this strain (Fig 2B), we reasoned that Δ*rpoH* cells are not able to compensate for the lack of DnaKJ chaperoning function at 30°C and 22°C. Indeed, we observed a strong increase in recovered protein aggregates after depletion of DnaKJ in the Δ*rpoH* background (Fig 2D). By contrast, the DnaKJ depletion strain containing the *rpoH(Q202R)* mutation, which shows intermediate σ^32^-dependent promoter activity (Fig 2B and 2C), behaved similarly to DnaKJ-depleted cells with wild type σ^32^ and did not show significant protein aggregation (Fig 2D). In summary, our data demonstrate that partial and, at low temperatures, complete loss of σ^32^ activity can bypass the lethality of DnaKJ depletion. These data suggest that DnaKJ’s regulatory function on σ^32^ is essential for survival in the absence of stress, while its chaperoning function is redundant and can be compensated by the upregulation of other HSPs.

We also examined σ^32^-dependent gene expression in the strains harboring mutations in *hslU*, *rpoD* or *rpoB* either by LacZ-assays or qRT-PCR. Interestingly, as for cells harboring mutations in *rpoH*, in these strains σ^32^-dependent transcription was also notably reduced after 6 and 9 hours of DnaKJ depletion (Fig 2B), suggesting that all suppressor mutations identified in the screen act through the same pathway.

### A gain-of-function mutation in *hslU* induces σ^32^ degradation and restores growth of DnaKJ-depleted cells

**Fig 3:**
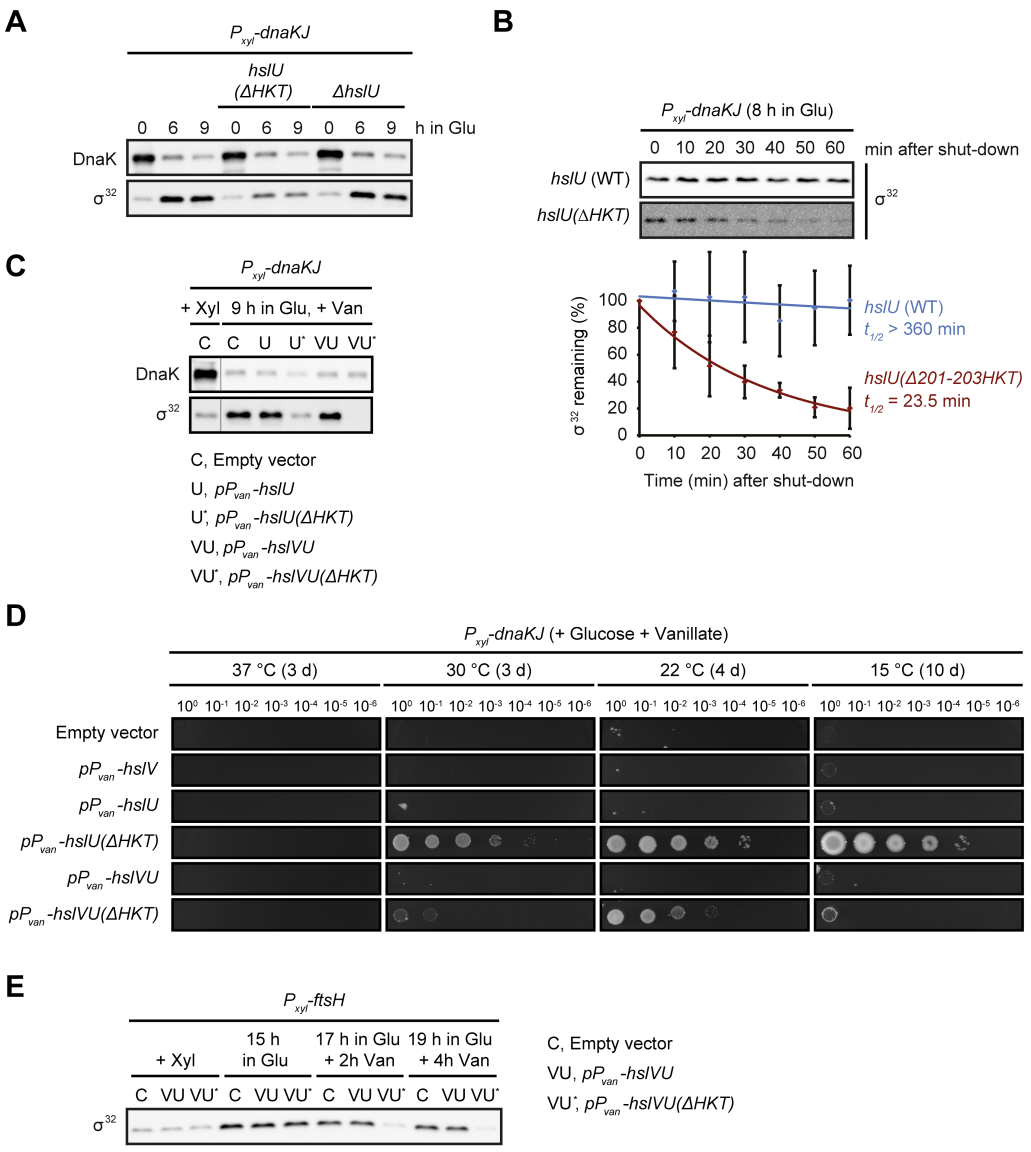
A mutation in the HslUV protease induces fast FtsH-independent degradation of σ^32^. (A) DnaK and σ^32^ steady-state levels during the course of DnaKJ depletion in an otherwise wild type background and in the *hslU(Δ201-203HKT)* and Δ*hslU* mutant backgrounds. (B) *In vivo* degradation assays showing σ^32^ stability after 8 h of DnaKJ depletion in the *hslU* wild type and the *hslU(Δ201-203HKT)* background. Quantifications are shown as the means of independent triplicates. Error bars represent standard deviations. (C) Western blot showing DnaK and σ^32^ steady-state levels of the DnaKJ depletion strain containing either the empty vector pJS14 or pJS14-based plasmids allowing for the vanillate-dependent overexpression of *hslU*, *hslU(Δ201-203HKT)*, *hslVU* or *hslVU(Δ201-203HKT)*. σ^32^ protein levels are shown for non-depleting non-overexpressing conditions (+ Xyl) and after nine hours of DnaKJ-depletion under overexpressing conditions (9 h in Glu, + Van). (D) Spot colony formation assay of the same strains as in (C) with addition of a strain containing the plasmid pJS14-*P*_*vanA*_*-hslV*. Cells were spotted on PYE agar under conditions depleting DnaKJ and driving overexpression from the high copy plasmids (+ Glucose + Vanillate). See S7 Fig for spot colony formation assays under non-depleting conditions. (E) σ^32^ steady-state levels before (+ Xyl) and after depletion of FtsH (+ Glu) in strains harboring empty pJS14, pJS14-*P*_*vanA*_*-hslVU* and pJS14-*P*_*vanA*_*-hslVU(Δ201-203HKT)*. After 15 hours of FtsH depletion overproduction of the HslUV variants was induced under continuous conditions restricting FtsH production (+ Glu + Van).

How does a mutation in *hslU* affect σ^32^-dependent gene expression? To test if growth restoration in DnaKJ-depleted cells harboring the *hslU(Δ201-203HKT)* allele was due to loss of function of the protease HslUV, we analyzed growth and *P*_*dnaKJ*_*-lacZ* expression following DnaKJ depletion in a Δ*hslU* strain lacking the HslUV unfoldase subunit HslU. However, unlike the *hslU(Δ201-203HKT)* suppressor mutation, this Δ*hslU* mutation neither reduced σ^32^-dependent gene expression nor restored growth in DnaKJ-depleted cells (Fig 2A and 2B). To test the possibility that the *hslU(Δ201-203HKT)* mutation affects σ^32^ degradation in DnaKJ-depleted cells, we measured σ^32^ steady-state levels and stability. During DnaKJ depletion σ^32^ levels significantly increase over the course of depletion, and deletion of the entire *hslU* gene has no effect on this observed increase. However, during DnaK depletion in the *hslU(Δ201-203HKT)* mutant, σ^32^ levels only slightly increase and remain low throughout (Fig 3A). Consistent with these data, after 8 h of DnaKJ depletion the half-life of σ^32^ was strongly decreased in the *hslU(Δ201-203HKT)* mutant (t_1/2_ ≈ 23.5 min), compared to the pronounced stabilization (t_1/2_ > 360 min) in the parental DnaKJ-depletion strain (Fig 3B). We next asked if overexpression of *hslU(Δ201-203HKT)* would also lead to reduced σ^32^ levels and restoration of colony formation in DnaKJ depleted cells. Western blots and spot assays confirmed reduction of σ^32^ levels and restored colony formation (Fig 3C and 3D). Furthermore, σ^32^ levels were reduced beyond detection when co-overexpressing *hslU(Δ201-203HKT)* along with *hslV*, encoding the proteolytic subunit of the protease complex. This stronger reduction could account for the fact that these cells could not grow at 30°C in the absence of DnaKJ but we cannot yet explain why they do not grow at 15°C. It is well established that FtsH is the protease normally degrading σ^32^ [3,4], therefore to investigate whether FtsH was required for the *hslU(Δ201-203HKT)*-dependent destabilization of σ^32^, we overexpressed *hslVU(Δ201-203HKT)* in cells depleted for FtsH. On its own, FtsH depletion leads to a strong stabilization of σ^32^, as expected (Fig 3E). In contrast, overexpression of *hslVU(Δ201-203HKT)* completely counteracted this stabilization (Fig 3E), demonstrating that *hslU(Δ201-203HKT)* destabilizes σ^32^ independently of FtsH. Furthermore, although the *hslU(Δ201-203HKT)* mutant readily destabilizes σ^32^, overexpression of wild type *hslU* alone or in conjunction with *hslV* had no effect on σ^32^ levels and could not restore growth in the absence of DnaKJ (Fig 3C–3E, S7 Fig). Based on these data and the fact that the *Δ201-203HKT* mutation is located in the substrate recognition region of HslU (S2 Fig), we propose that this mutation is a gain-of-function mutation rendering HslUV capable of degrading σ^32^. Importantly, we neither observed increased degradation of the two unstable proteins CtrA and FtsZ nor the stable protein RpoB in the *hslU(Δ201-203HKT)* mutant strain (S6 Fig). Therefore, while these data indicate that the HslVU(Δ201-203HKT) mutant does not affect the majority of proteins, at this point we cannot say whether the effect of the mutation is restricted to σ^32^ or if the degradation of other proteins is affected as well.

### Outcompetition of σ^32^ by σ^70^ for binding to the RNAP rescues DnaKJ-depleted cells

**Fig 4:**
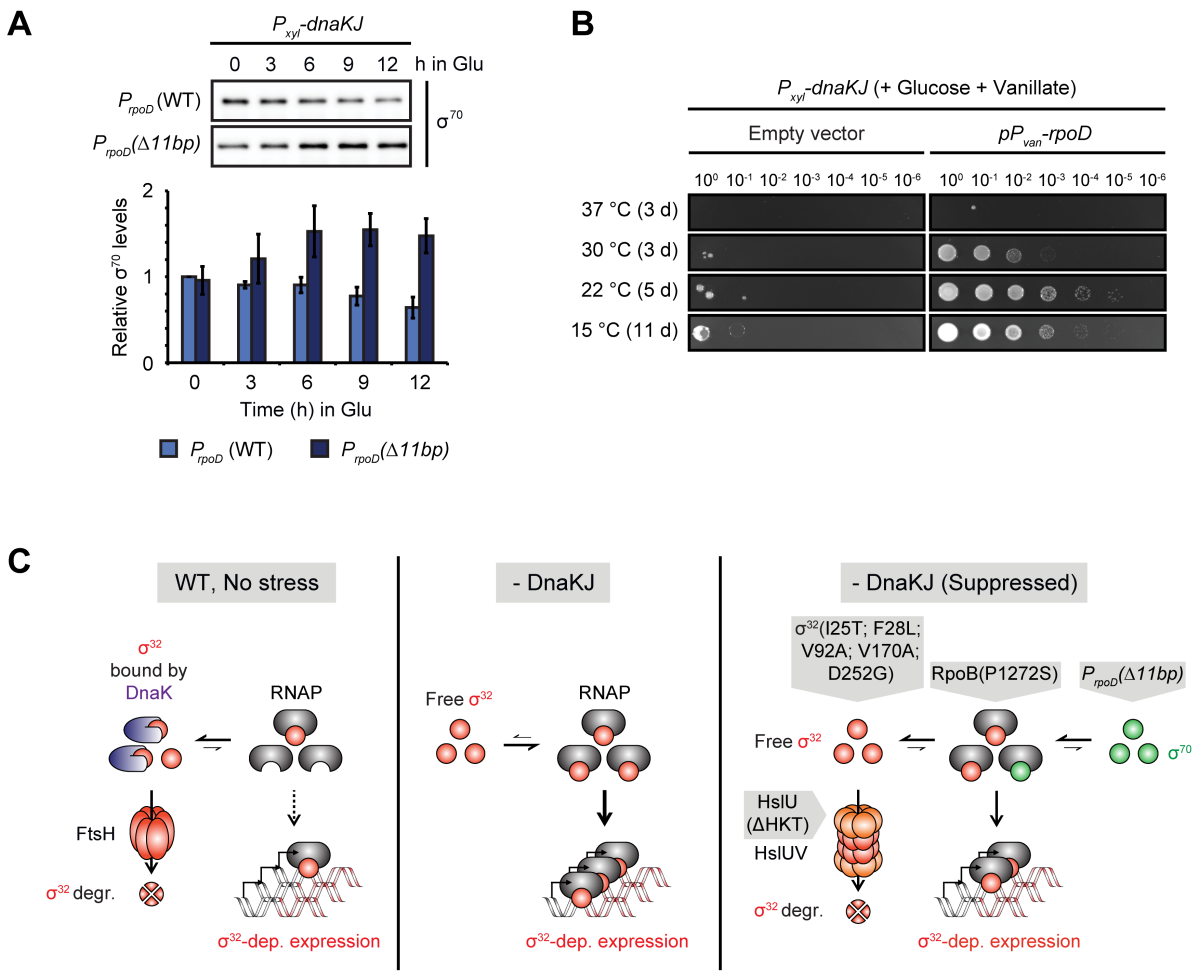
Increased levels of σ^70^ reduce σ^32^-dependent expression. (A) σ^70^ steady-state levels during DnaKJ depletion in the *P*_*rpoD*_*(Δ11bp)* background compared to the unsuppressed DnaKJ depletion background, *P*_*rpoD*_ (WT). Quantifications are shown as the means of independent triplicates. Error bars represent standard deviations. (B) Spot assay of the DnaKJ depletion strain harboring either the empty vector pBVMCS-2 or the pBVMCS-2-*P*_*vanA*_*-rpoD* plasmid on PYE agar plates containing glucose. Vanillate induces *rpoD* expression. (C) Model illustrating how the different suppressor mutations reduce σ^32^ activity. In the wild type (WT) DnaKJ inactivates σ^32^ and primes it for degradation by the protease FtsH, thus inhibiting expression from the σ^32^-regulon. Depletion of DnaKJ leads to stabilization and activation of σ^32^, which is now free to associate with the RNAP and to induce σ^32^-dependent gene expression. Mutations restoring growth in the absence of DnaKJ lower σ^32^ activity, either by loss of function amino acid exchanges in the heat shock sigma factor itself, by inducing its degradation through the protease HslUV, by lowering the affinity of RNAP for σ^32^ or by increasing the levels of σ^70^ that outcompetes σ^32^ for binding to the RNAP core.

Next, we addressed the molecular basis of how the 11 bp deletion in the promoter region of *rpoD* restores growth in DnaKJ-depleted cells. To this end, we tested if the mutation alters σ^70^ levels during the course of DnaKJ depletion (Fig 4A). Initially, the amount of σ^70^ was indistinguishable between the mutant and the parental strain. However, during the course of depletion, σ^70^-levels decreased to ~60% in the wild type background while they increased to ~150% in the *rpoD* promoter mutant. To test whether upregulation of σ^70^ is sufficient to restore growth of DnaKJ-depleted cells, we ectopically overexpressed *rpoD* in the DnaKJ depletion strain. Indeed, during DnaKJ depletion and *rpoD* overexpression colonies formed at low and intermediate temperatures (Fig 4B). Based on previously proposed sigma competition models [29,30], we suggest that increased levels of σ^70^ may outcompete σ^32^ for binding to the RNAP core. In accordance with this model, the suppressor mutation in the *rpoD* promoter led to reduced σ^32^ occupancy of the σ^32^-dependent *hslVU* promoter compared to the parental DnaKJ depletion strain, without significantly affecting σ^32^ levels (Fig 2C, S4 Fig). Further, the *rpoB(P1272S)* suppressor mutation we identified is located in a region known to be required for interaction with sigma factors (S2 Fig) [31]. Thus, this mutation might impair σ^32^ association with the RNAP core, a hypothesis supported by the observation that *hslVU* promoter occupancy by σ^32^ was reduced in cells harboring the *rpoB(P1272S)* allele (Fig 2C).

Altogether our data clearly demonstrate that attenuation of σ^32^ activity, whether by mutation or deletion of the *rpoH* gene, by increasing σ^32^ degradation, by outcompeting σ^32^ for binding to the RNAP, or by reducing the affinity of the RNAP towards σ^32^, can bypass the growth defect of DnaKJ depleted cells (Fig 4C).

### Overproduction of active σ^32^ reduces the growth rate

Our data strongly suggest that DnaKJ is essential to prevent inappropriate induction of σ^32^-dependent genes or high levels of the sigma factor itself, which could be detrimental to the proper functioning of the cell. To test this hypothesis more directly, we next analyzed the effects of *rpoH* overexpression on the growth rates.

**Fig 5:**
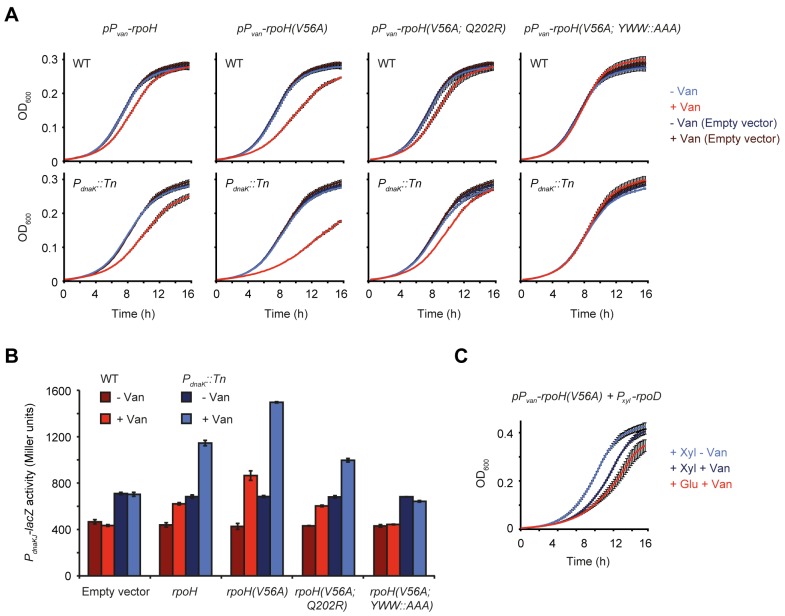
σ^32^ inhibits growth in a dose-dependent manner. (A) Growth curves of the wild type and *P*_*dnaK*_::*Tn* strain harboring either the empty vector pJS14 or a plasmid for the vanillate-inducible overexpression of *rpoH*, *rpoH(V56A)*, *rpoH(V56A; Q202R*) or *rpoH(V56A; YWW*::*AAA)*. Growth was monitored in the absence or presence of vanillate. (B) LacZ assays monitoring σ^32^-dependent expression from the *dnaKJ* promoter region on the reporter plasmid pDel1 in the otherwise same strain backgrounds and under the same conditions as shown in (A). (C) Growth curves of the *P*_*dnaK*_::*Tn* strain harboring a plasmid allowing for combined overexpression of *rpoD* and *rpoH*. Vanillate induces the expression of *rpoH*, xylose induces the expression of *rpoD*. All experiments were performed as independent duplicates. Error bars represent standard deviations.

Overexpression of *rpoH* led to a noticeable decrease in growth rate, particularly in a strain containing a transposon insertion in the *dnaKJ* promoter (*P*_*dnaK*_::*Tn*) (Fig 5A), which is deficient in upregulating *dnaKJ* upon σ^32^ activation leading to a more persistent induction of the heat shock response [23] (Fig 5B). The growth defect was further amplified when overproducing a σ^32^ variant containing the amino acid substitution V56A, which corresponds to the I54A substitution in *E*. *coli* that allows σ^32^ to escape FtsH-dependent degradation [32,33]. Although overexpression of *rpoH(V56A)* clearly induced heat shock gene expression more strongly than wild type *rpoH*, we did not observe stabilization of the mutant variant (S8 Fig), suggesting that in *C*. *crescentus* this mutation increases σ^32^ activity by another mechanism. The growth inhibition induced by σ^32^(V56A) overproduction was clearly reduced by introducing the Q202R suppressor mutation into σ^32^(V56A). Similarly, disruption of σ^32^(V56A) transcriptional activation by exchanging the three amino acids Y106, W109 and W110 responsible for promoter melting [34–37] against alanines (*YWW*::*AAA*) abolished heat shock response induction and completely restored normal growth (Fig 5A and 5B). Measurement of σ^32^ steady state levels after vanillate-dependent induction confirmed that all variants were expressed at similar levels (S8 Fig). Finally, the deleterious effect of elevated σ^32^(V56A) levels could also be counteracted by overproducing σ^70^ (Fig 5C), supporting the hypothesis that σ^70^ can rescue DnaKJ-depleted cells by outcompeting σ^32^ for binding to the RNAP, thus reducing σ^32^-dependent transcription. In summary, these data show that σ^32^ inhibits growth in a dose-dependent manner and does so, albeit to a lower degree, also in cells harboring DnaKJ.

### σ^32^ globally reprograms gene expression

**Fig 6:**
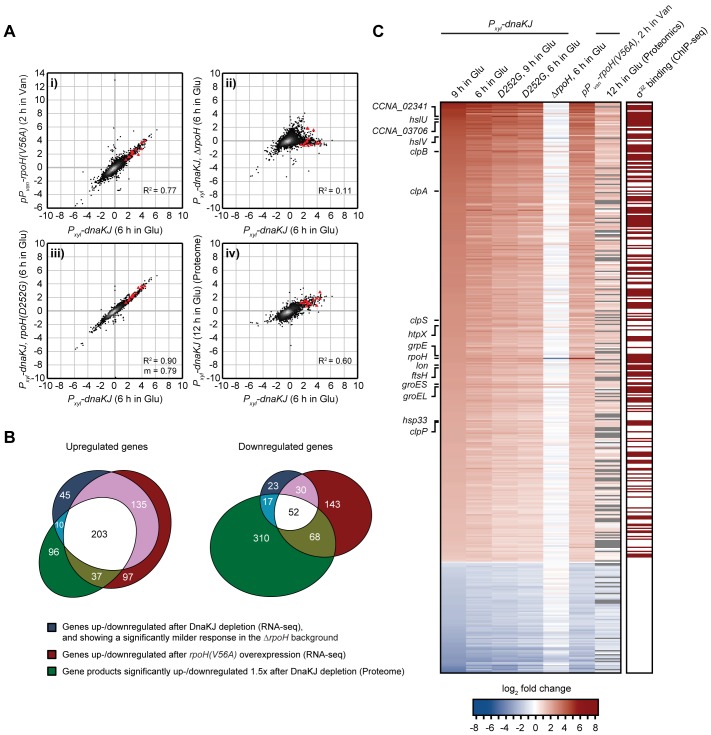
σ^32^ activation leads to global changes in gene expression. (A) Gene scatter plots comparing gene expression changes induced by 6 hours of DnaKJ depletion with: i) gene expression changes induced by 2 hours of σ^32^(V56A)-overproduction in the *P*_*dnaK*_::*Tn* background, ii) gene expression changes in DnaKJ-depleted cells (6 hours) containing the Δ*rpoH* mutation iii) gene expression changes in DnaKJ-depleted cells (6 hours) containing the *rpoH(D252G)* mutation and iv) protein fold changes after 12 h of DnaKJ depletion. All experiments were performed at 30°C. Red triangles represent highly conserved proteases and chaperones that are highlighted in the heat map in (C). m, as the slope of the linear regression is shown when the corresponding R^2^ designates a close fit of the data points and allows for a direct comparison of gene induction in samples of the same strain background. See S9 Fig for additional gene expression comparisons. (B) Venn diagrams showing groups of up- or downregulated genes that meet the criteria described in the figure. The intersection between the blue and red gene groups (shown in white and pink) was defined as the group of genes affected by σ^32^-activity. The three-way intersection (shown in white) represents genes of which the corresponding protein is similarly up- or downregulated due to σ^32^-activity. (C) Heat map showing mRNA and corresponding protein fold changes of σ^32^-dependent genes identified in (B) (blue and red gene group intersections) in the different genetic backgrounds and growth conditions shown. The right column indicates with a dark red box whether a gene shows a ChIP-seq signal for σ^32^-enrichment in the promoter region as inferred from previously published ChIP-seq data [38]. Gray bars in the column labeled "12 h in Glu (Proteomics)" indicate that the corresponding protein could not be detected.

To better understand the inhibitory effect of σ^32^ on growth we monitored transcriptome changes induced by σ^32^ activation through RNA-sequencing. The transcriptional profile of DnaKJ-depleted cells harboring wild type *rpoH* correlated well with σ^32^(V56A) overproducing cells (R^2^ = 0.77) (Fig 6A). By contrast, in DnaKJ-depleted cells containing the Δ*rpoH* deletion this correlation was largely lost (R^2^ = 0.11), confirming that transcriptional changes after DnaKJ depletion are mainly due to increased σ^32^ activity. In the DnaKJ depletion strain containing the *rpoH(D252G)* mutation, gene expression changes were similar to the parental DnaKJ depletion strain, however less pronounced (Fig 6A and 6C; S9 Fig). Genes that were >2-fold up- or downregulated both upon DnaKJ depletion and upon σ^32^(V56A) overexpression and that showed a significantly milder or no response (> 2-fold) in the Δ*rpoH* background were classified as σ^32^-regulated genes. We found in total 338 up- and 82 downregulated genes, belonging to this group (Fig 6B and 6C).

Integration of our transcriptomics data with existing *C*. *crescentus* σ^32^-ChIPseq data [38] revealed that 181 of the 338 upregulated genes harbor an upstream ChIP-seq signal for σ^32^-enrichment or are in an operon with such a gene (Fig 6C). These 181 directly upregulated genes include most heat shock genes encoding highly conserved proteases and chaperones, but also many metabolic genes as well as genes involved in small molecule membrane transport (S9 Fig). Likewise, the group of downregulated genes included genes involved in metabolism and membrane transport, but also several genes encoding cell cycle regulators and factors involved in cellular development (S9 Fig). Hence, σ^32^ induction leads to wide transcriptomic changes that go far beyond the upregulation of chaperones and proteases.

### σ^32^ induction re-allocates cellular resources from proliferative to maintenance functions

**Fig 7:**
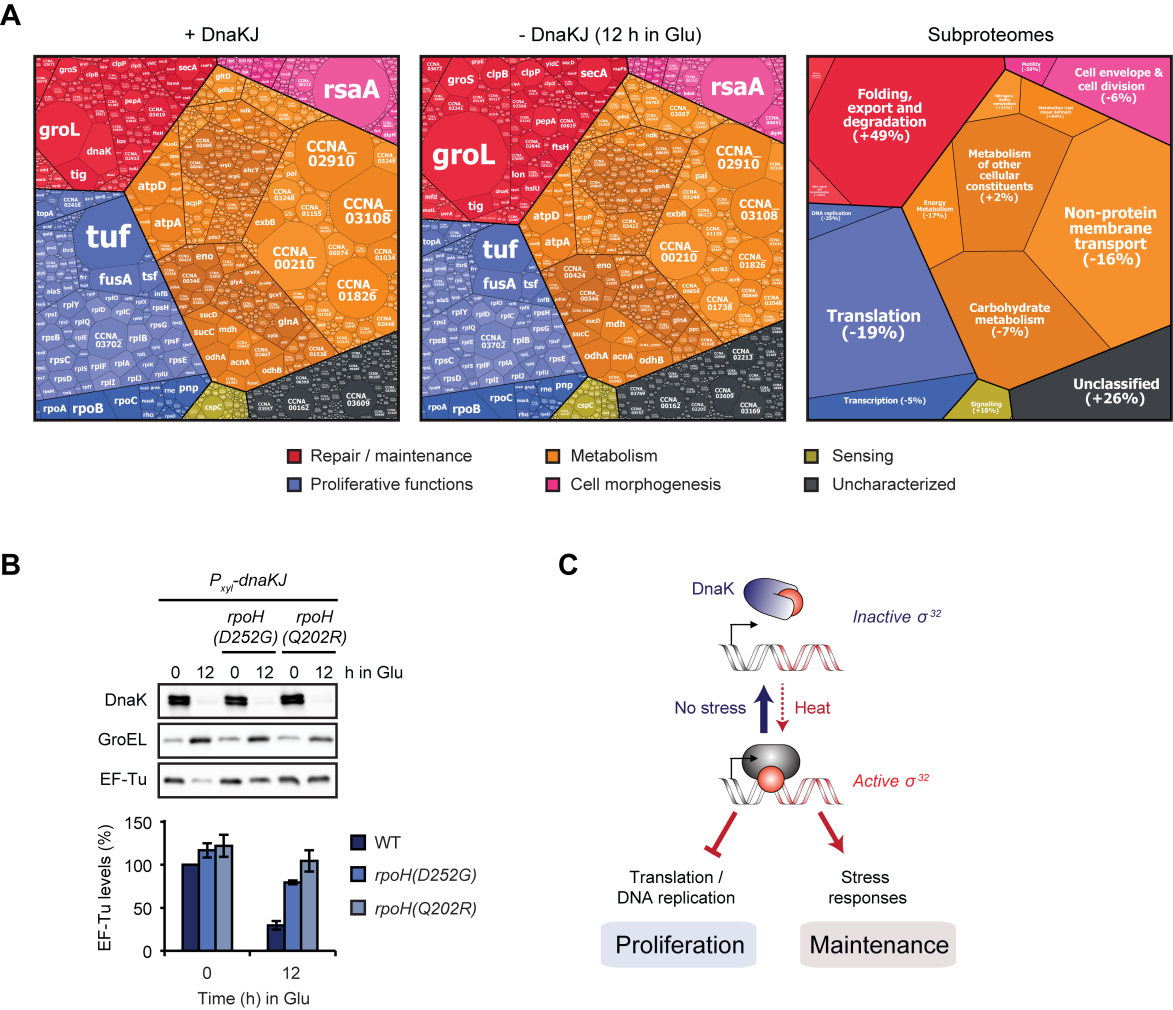
σ^32^-dependent re-allocation of the molecular investment between mass fractions of the proteome. (A) Proteomaps showing the amino acid investment of cells into different cellular processes of *dnaKJ*-expressing and DnaKJ-depleted cells (12 h in Glu). The treemap on the right shows the functional categories of subproteomes that are represented in the map and the percentual changes of their mass fraction after 12 h of DnaKJ-depletion compared to undepleted cells. The size of each area of the mosaic reflects the abundance of the corresponding protein. See S10 Fig for an additional treemap. (B) Western blots showing DnaK, GroEL and EF-Tu steady state levels in the presence of DnaKJ and after 12 h of DnaKJ depletion in different genetic backgrounds. Quantifications are shown as the means of independent duplicates. Error bars represent standard deviations. (C) Model illustrating how the interaction between DnaK and σ^32^ reciprocally controls proliferative and maintenance functions. σ^32^ upregulates stress pathways while downregulating global protein synthesis and DNA replication. Growth and proliferation under non-stress conditions strictly depends on DnaK, which keeps σ^32^ activity low. Under heat stress conditions σ^32^ is released to re-allocate resources from proliferative to maintenance functions.

To gain a more complete view on the cellular effects mediated by σ^32^ induction following DnaKJ depletion, we compared the proteome in DnaKJ-depleted cells to the non-depleted control by quantitative proteomics using a tandem mass tag (TMT) isobaric labeling approach. We identified 346 proteins that were at least 1.5-fold upregulated (p **≤** 0.05) (S9 Fig). For 203 of these proteins the corresponding transcripts were also upregulated in a σ^32^-dependent manner (Fig 6B and 6C). In the case of the 447 downregulated proteins (> 1.5-fold, p **≤** 0.05), less than 12% of the corresponding genes were transcriptionally downregulated. To investigate energetic investment into the different functional categories of the proteome in DnaKJ-depleted and non-depleted cells, we compared the relative abundances of all analyzed proteins using proteomaps [39]. Depletion of DnaKJ induced large-scale changes of proteome fractions dedicated to maintenance, metabolism and proliferative processes. More specifically, the mass fraction of the proteome dedicated to folding, export and degradation increased by 49% due to the strong upregulation of chaperones and proteases, particularly due to the highly abundant GroEL/ES chaperone (Fig 7A and 7B, S10 Fig). By contrast, the majority of differentially regulated abundant proteins involved in protein translation were downregulated. The downregulation of this group of proteins likely accounts for the decrease in total protein synthesis that we have previously observed in DnaKJ depleted cells [23]. In particular, levels of the highly abundant translation factor EF-Tu (encoded by *tuf*), and the small ribosomal protein RpsF were notably reduced (by 45% or 44%, respectively) (Fig 7A). Importantly, compromised activity of σ^32^ in the *rpoH(D252G)* and *rpoH(Q202R)* backgrounds restored EF-Tu levels in DnaKJ-depleted cells (Fig 7B), demonstrating that the observed downregulation depends on σ^32^.

Taken together our results demonstrate that induction of σ^32^ in DnaKJ-depleted cells induces wide proteome changes and a reorganization of cellular functions. While the major proteome fractions dedicated to repair and maintenance functions were upregulated, major subproteomes required for the growth-promoting functions of protein translation, DNA replication and for certain metabolic processes were downregulated.

## Discussion

In this study we have analyzed in detail the essential function of DnaK in an α-proteobacterium in which the chaperone is strictly required for growth at all temperatures. Our study demonstrates that the reason for DnaK's essentiality under non-stress conditions is its role in repressing σ^32^ activity and in this way counteracting extensive reprogramming of global gene expression, with growth-inhibitory consequences (Fig 7C). The ancestral function of DnaK as a folding catalyst is required only at elevated temperatures. Under optimal conditions it is dispensable and its loss can be compensated by other chaperones and proteases. Our data highlight that gene essentiality can have multiple causes that differ depending on the growth condition; in the case of DnaK, two essential functions switch in a temperature-dependent manner.

Our conclusions regarding DnaK's essentiality are based on suppressor mutations that bypass the lethality of DnaK depletion and that all act by attenuating σ^32^ activity (Fig 4C). Besides *cis*-acting mutations in the *rpoH* gene that reduce σ^32^ activity, we also identified mutations in *rpoD*, *rpoB* and *hslU* that reduce σ^32^ activity *in trans*, providing important new insight into the regulatory pathways controlling σ^32^ activity. Most notably, a mutation in *hslU* led to increased σ^32^ degradation, and a mutation in the *rpoD* promoter resulting in higher levels of the housekeeping σ^70^ negatively affected σ^32^-dependent gene expression. HslUV is the most recently identified ATP-dependent bacterial protease [40]. Despite much biochemical and structural insight, the physiological roles of this protease remain poorly understood and only few confirmed substrates are known in *E*. *coli*. In *Caulobacter*, which has advanced as an important model for regulated proteolysis [41], no substrates have yet been identified for this protease. Past work proposed that HslUV together with the other cytosolic proteases might degrade σ^32^ in *E*. *coli* [42]. A follow-up study showed that purified *E*. *coli* HslUV degrades *E*. *coli* σ^32^ *in vitro* at elevated temperatures [43]. In our experiments, we observed that the *hslU(Δ201-203HKT)* mutation caused rapid FtsH-independent degradation of σ^32^. By contrast, we did not observe degradation of σ^32^ by wild type HslUV, indicating that removal of the three amino acids HKT in the substrate recognition region of HslU may alter substrate selectivity or alternatively, bypass the need for an accessory factor that is required to deliver σ^32^ to HslUV and might be present only under certain conditions. Future work will characterize the discovered HslU mutation in more detail and elucidate the role of wild type HslUV in σ^32^ degradation.

Our data also demonstrate that elevated levels of the housekeeping σ^70^ can negatively affect σ^32^-dependent gene expression, demonstrating that the two sigma factors are able to influence each other. In agreement with sigma-competition models [29,30], our data suggest that σ^70^ outcompetes σ^32^ for binding to the RNAP and in this way reduces σ^32^-dependent transcription. Previous work showed that under heat stress conditions σ^70^ is aggregation prone [44], which might further relieve competitive pressure on σ^32^ when necessary. While our data demonstrate that increased σ^70^ levels can inhibit σ^32^, it is possible that conversely high levels of active σ^32^ during heat shock or DnaK depletion inhibit σ^70^ from associating with the RNAP. A σ^32^-induced reduction of σ^70^-dependent transcription could contribute to the global gene expression changes that we observed in our RNA-sequencing experiments.

Mutations reducing σ^32^ activity have also been found to improve growth at low temperatures in a *dnaK* deletion mutant in *E*. *coli* [27]. In an analogous way, growth defects at physiological temperatures after loss of the cytoplasmic Hsp70s SSA1 and SSA2 in yeast can be suppressed by mutations that attenuate the functionality of the heat shock transcription factor HSF1 [45]. The emergence of regulatory interactions between Hsp70 homologs and heat shock response effectors during evolution has thus added a new layer of cellular dependency on a chaperone system whose main ancestral function as a folding catalyst is dispensable at optimal temperatures. Noticeably, in *Bacillus subtilis*, a member of the firmicutes, which does not possess σ^32^, deletion of DnaK has almost no observable consequences in the absence of stress [46]. It remains to be studied whether the deleterious effects of DnaK loss in other proteobacteria are linked to increased σ^32^ activity and global gene expression changes and how the functions of DnaK are utilized for survival in other bacterial groups.

Our data demonstrate that liberated σ^32^ activity shifts cellular resources from proliferative functions to cytoprotective functions. While this re-allocation leads to detrimental growth defects under favorable conditions, it could represent an effective way to protect stress sensitive processes and to re-allocate resources to active stress adaptation. In particular, nascent proteins are aggregation prone since incomplete polypeptides cannot yet fold into their native conformation and are threatened by unproductive interactions with other chains emerging from neighboring ribosomes [47]. Slowing down growth and global protein synthesis can provide advantageous conditions for protein folding when the chaperoning capacity is limited [13,48]. Furthermore, slowly growing or dormant cells are often resistant to certain stress conditions like antibiotic treatment [49].

## Materials and methods

Strains, plasmids, and primers used are listed in S1 Table. Plasmid and strain construction procedures are described in S1 Text.

### Bacterial growth conditions

*C*. *crescentus* cultures were grown in the complex medium PYE or the minimal medium M2 with the following supplements if required: 0.3% xylose, 0.2% glucose, 3% sucrose, 1 mM IPTG or 500 mM vanillate. Antibiotics were added in concentrations as described earlier [23]. Liquid cultures were routinely grown while shaking at 200 rpm at the desired temperatures and regularly diluted to assure growth in the exponential phase. To deplete proteins of interest, cells were washed two times with PYE by centrifugation (6000 g, 4 min) before resuspension in medium devoid of the inducer. Transductions were performed using ϕCr30 as described previously [50]. *E*. *coli* was grown for cloning purposes in LB supplemented with antibiotics as necessary at 37°C.

### Genetic screen for DnaKJ depletion suppressors

We performed the suppressor screen with strain ML2009 [23] a derivative of the xylose-dependent DnaKJ depletion strain SG400 [21], which contains a chloramphenicol resistance-conferring high copy plasmid carrying *P*_*vanA*_-*dnaA*. Shifting the cells from a xylose-containing to a glucose-containing medium results in a block of *dnaKJ* expression while addition of vanillate allows for the inducible overproduction of the DNA replication initiator DnaA. Overly high levels of DnaA are lethal and can be counteracted by increased degradation through the protease Lon which is upregulated as part of the heat shock response after depletion of DnaKJ [23]. By overproducing DnaA in DnaKJ-depleted cells, we intended to produce selective pressure against restoration of DnaKJ levels through mutations in the xylose-dependent promoter or *xylR* which would fully abolish heat shock gene induction and thus decrease degradation of DnaA.

ML2009 cells were depleted of DnaKJ for five hours without overproduction of DnaA in PYE liquid medium containing glucose and chloramphenicol. 100 μL of log-phase culture (OD_600_ of 0.1–0.4) were plated on PYE agar plates containing glucose/chloramphenicol/vanillate to repress DnaKJ synthesis and to induce overexpression of *dnaA*. Colonies appearing after three, four and five days of incubation at 30°C were restreaked on PYE plates supplemented with either xylose/chloramphenicol or glucose/chloramphenicol/vanillate. Cells grown on the latter medium were restreaked on plates containing xylose/chloramphenicol/vanillate to check for continued lethality of *dnaA* overexpression in the presence of DnaKJ and to confirm the absence of mutations reducing DnaA functionality. Clones incapable of growing on xylose/chloramphenicol/vanillate were cultured overnight in liquid PYE medium supplemented with either xylose/chloramphenicol or glucose/chloramphenicol/vanillate. Cells grown under DnaKJ producing (PYE supplemented with xylose/chloramphenicol) as well as DnaKJ-depleting and DnaA-overproducing conditions (PYE supplemented with glucose/vanillate/chloramphenicol) were frozen and tested for the absence of DnaK by western blot. The majority of clones which arose after three or four days of incubation on the initial plate had restored DnaK levels to varying degrees, indicating that these clones harbored mutations in the xylose-inducible promoter. By contrast, in most clones that appeared after five days, DnaK levels were as low as in the parental strain following 24 hours of depletion, indicating that these clones have acquired mutations that allow them to grow in the absence of DnaK. Suppressors in *rpoH* were identified by conventional sequencing of the *rpoH* gene. The other suppressor clones were verified for the absence of mutations in the gene encoding the XylR repressor or in *P*_*xyl*_ upstream of the *dnaKJ* operon and then subjected to whole genome sequencing. In addition to mutations in *rpoH*, *rpoB*, *rpoD* and *hslU*, we also identified five mutations in other genes (S2 Table). Because of genetic instability and difficulties to re-introduce these mutations into the DnaKJ depletion strain, these clones were excluded from further analysis.

### Whole genome sequencing

Whole genome sequencing was performed by GENEWIZ, South Plainfield, NJ using an Illumina Nextera XT DNA prep workflow and sequencing on MiSeq with 2x150bp configuration. Mapping to the *Caulobacter crescentus* NA1000 (NC_011916) reference sequence was performed for each of the samples, after which detection of SNPs/INDELs was conducted.

### Microscopy

Cells analyzed by phase-contrast microscopy were fixed with 1% formaldehyde immediately after sampling and stored at 4°C. Prior to analysis the cells were mounted on PYE 1% agarose pads. Phase contrast images were obtained using a T*i* eclipse inverted research microscope with a 100x/1.45 NA objective (Nikon). The images were processed with Fiji (ImageJ).

### Flow cytometry

Flow cytometry samples were prepared as previously described [51] and analyzed with the BD LSRFortessa (BD Biosciences) flow cytometer. For each sample 30000 cells were analyzed. The experiments were performed in biological replicates and representative results are shown. The obtained data were analyzed and processed with FlowJo.

### Western blot analysis

Sample preparation and western blot analysis was performed as previously described [52]. Equal loading of total protein and the quality of the transfer was assessed through protein visualization using the TCE in-gel method [53]. The following primary antibodies were used in appropriate dilutions: α-DnaK (this study), α-DnaA [54], α-GroEL (kindly provided by S. L. Gomes), α-CtrA (kindly provided by M. Laub), α-FtsZ (kindly provided by M. Thanbichler), α-σ^32^ (clone 3NR3, BioLegend Cat. No. 663402 (used in experiment for Fig 3B) and an antibody kindly provided by F. Narberhaus (used in all other experiments), α-σ^70^ (clone 2G10, BioLegend Cat. No. 663203), α-RpoB (clone 8RB13, Biolegend Cat. No. 663903) and α-EF-Tu (clone 900, Hycult Cat. No. HM6010). The primary antibodies were detected using secondary HRP-conjugated antibodies. SuperSignal Femto West (Thermo Scientific) served as detection reagent. The blots were scanned using the Chemidoc (Bio-Rad) system. Relative band intensities were quantified with the Bio-Rad Image Lab software and images were processed with Fiji (ImageJ).

### DnaK antibody production

His_6_-DnaK was expressed from the pML375-*dnaK* plasmid and purified by Ni-NTA affinity chromatography as described previously [55]. The purified His_6_-DnaK was used to generate rabbit polyclonal antisera against DnaK (Davids Biotechnologie GmbH).

### Spot colony formation assays

Cell cultures with an OD_600_ corresponding to the exponential phase of wild type cells (OD_600_ 0.1–0.4) in PYE were diluted to OD_600_ 0.05 and serially diluted 1:10 before 2 μL of each dilution were spotted on PYE rich or M2 minimal medium agar plates.

### Growth monitoring using plate readers

Cultures were diluted to an OD_600_ of 0.025 before being added as 100 μL volumes into sterile 96 well plates. Cells were cultured shaking at 30°C and measured every 10 min using either an infinite 200 Pro (Tecan) (linear shaking amplitude 3.5 mm) or a SpectraMax i3x (Molecular Devices) (linear shake intensity set to high) plate reader.

### *In vivo* protein aggregation assay

The *in vivo* aggregation protocol was adapted from previously published procedures [19,56,57]. About 40 OD_600_ units of exponential cells were rapidly cooled in an ice-water bath and pelleted (6000 g, 10 min, 4°C). All following steps were performed at 4°C and buffers used during or after cell lysis were supplemented with cOmplete ULTRA protease inhibitor cocktail (Sigma-Aldrich). The pellets were washed once in buffer A (50 mM Tris/HCl pH8.0, 150 mM NaCl) and resuspended in 4 mL buffer A supplemented with 12 U/mL benzonase (Merck Millipore Cat. No. 70746). Cells were lysed by two passages at 1000 psi in a FRENCH Press using a Manual-Fill Mini-Cell (FA-003) (Thermo Spectronic). Lysates were centrifuged (5000 g, 10 min) two times in order to remove intact cells. The protein concentration of the cleared lysate was measured by Bradford assay and 3 mL were centrifuged (20000 g, 20 min) to pellet the insoluble protein fraction. The supernatant was discarded, the pellets were resuspended in 300 μL buffer A and subsequently sonicated in a Bioruptor (Diagenode) (set to high, 1 cycle for 30 s, 4°C) followed by a centrifugation (20000 g, 20 min) for washing. This procedure was repeated two more times using buffer A supplemented with 1% (v/v) Triton X-100 with an incubation of 1 h on ice with regular vortexing prior to the sonication, before a last wash step identical to the first one was performed. The protein pellet was resuspended in 100 μL buffer A by sonication (set to high, 1 cycle for 30 s, 4°C), supplemented with 5x SDS loading buffer and heated to 95°C for 10 min. Protein fractions were normalized to each other according to the concentration measurements of the Bradford assay by addition of 1x SDS loading dye.

### β-galactosidase activity measurements

Cells containing the *P*_*dnaKJ*_-lacZ (pDel1) reporter were harvested from exponential phase (OD_600_ 0.1–0.4) PYE (supplemented with tetracycline for maintenance of the reporter plasmid) liquid cultures grown shaking at 30°C. β-galactosidase activity was measured using the standard protocol [58].

### Quantitative RT-PCR

RNA was isolated from log phase bacterial cultures grown shaking at 30°C using the RNeasy mini kit (Qiagen). Equal amounts of RNA were reverse transcribed into cDNA using the iScript cDNA synthesis kit (Bio-rad). The obtained cDNA served as template in a real-time PCR reaction using the iTaq universal SYBR Green Supermix (Bio-rad). For the analysis of 16S rRNA or *hslU* mRNA levels in each sample, the primer pairs RT_16SFor/RT_16SRev and OFS399/OFS400 were used, respectively. The analysis was performed using a StepOnePlus real-time PCR system (AppliedBiosystems, Foster City, CA) in standard run mode. For each reaction a dissociation curve was run after completion to rule out detection of primer dimerization or amplification artifacts. Gene expression profiles of *hslU* were normalized against 16S rRNA levels as endogenous control. Relative expression levels were calculated employing the comparative Ct method.

### Quantitative chromatin immunoprecipitation (qChIP) assays

Samples were prepared according to a previously published protocol [59] with the modifications to the protocol and the subsequent qRT-PCR as described in Heinrich *et al*. 2016 [52]. For immunoprecipitation of σ^32^-DNA complexes an α-σ^32^ antibody (kindly provided by F. Narberhaus) was applied in a 1:400 dilution. For measuring promoter occupancy by σ^32^, qRT-PCR assays were performed with primers OFS984/OFS985 amplifying a 100 bp region encompassing the heat shock-inducible promoter of the *hslVU* operon, corresponding to a σ^32^-binding site according to Haakonsen *et al*. 2015 [38].

### *In vivo* degradation assay

*In vivo* protein degradation rate measurements were performed as previously described [23]. Chloramphenicol was used at a concentration of 100 μg/mL to shut down protein synthesis.

### RNA sequencing, transcriptome and ChIP-seq data analysis

RNA was extracted from cell pellets using the RNeasy mini kit (Qiagen). RNA-sequencing was performed by GENEWIZ, South Plainfield, NJ. Fold changes were calculated as the ratio between the normalized expression values of the respective DnaKJ depletion samples or the σ^32^(V56A) overexpression and the non-depleted *P*_*xyl*_*-dnaKJ* reference sample. Transcripts were considered differentially regulated after DnaKJ depletion in the WT background when they were at least twofold up- or downregulated after six and nine hours of depletion (S3 Table). Gene expression data are available at the Gene Expression Omnibus repository: GSE102372.

For the identification of putative direct transcriptional targets of σ^32^ we used published ChIP-seq data from Haakonsen *et al*. 2015 [38] (GSM1906341). Peaks were defined where the read density remained above 10, assigned to the position where their maximum value was found (+/- 10 bp due to binning of the original data), and summed to determine the peak area. We then searched for ORFs with a translation start site within 200 bp up- and 60 bp downstream of the peak maximum. ORFs were considered to be in an operon if specified by Schrader *et al*. [60] in which case all peaks potentially affecting transcription of the ORF were considered.

### Quantitative proteomics

For quantitative proteomics experiments 100 mL cultures of SG400 cells grown in PYE xylose or in PYE glucose for 12 h to an OD_600_ between 0.1 and 0.4 were rapidly cooled in an ice water bath. The cultures were pelleted (6000 g, 10 min, 4°C) and washed once with ice-cold ddH_2_O. Each condition was cultured as independent biological triplicates. The cell pellets were kept at -80°C before analysis. Protein digestion, TMT10 plex isobaric labeling and the mass spectrometrical analysis was performed by the Clinical Proteomics Mass Spectrometry facility, Karolinska Institute/Karolinska University Hospital/Science for Life Laboratory. Sample protein abundances were normalized to portions of 100% for each sample and each protein quantity mean of the experimental sample was divided by the mean of the control sample. The statistical significance of the log_2_-fold changes was assessed using *p*-values obtained based on Student’s t-test (S3 Table). Proteins were considered differentially regulated after DnaKJ depletion when they were at least 1.5-fold up- or downregulated.

### Proteomaps

Proteomaps were generated as previously described [39,61]. Functional categorizations were manually assigned to the proteins identified through mass spectrometry using KEGG pathway maps, gene ontology (GO—biological process) as basis and by literature search (S3 Table).

## Supporting information

S1 Fig The DnaKJ chaperone system is required for growth on minimal medium and both chaperones are essential in isolation. (A) Colony growth of wild type cells and the DnaKJ depletion strain grown for the indicated time at different growth temperatures on M2 minimal medium plates containing either glucose or xylose. (B) Colony growth of a DnaKJ depletion strain harboring an additional copy of *dnaJ* under the control of the chromosomal *P*_*vanA*_ promoter at different growth temperatures. Growth was assessed under conditions promoting expression of *dnaKJ* and *dnaJ* (+ Xylose + Vanillate) and under conditions only expressing *dnaJ* (+ Glucose + Vanillate). (C) Spot colony formation assay of a vanillate-dependent DnaJ depletion strain at different temperatures under non-depleting (+ Vanillate) and depleting (- Vanillate) conditions.
(EPS)

S2 Fig Graphical summary of the locations of the identified suppressor mutations. Amino acid sequence changes are shown on the protein domain structures of σ^32^, RpoB and HslU while changes affecting a non-coding sequence are shown for the DNA sequence spanning the promoter region of *rpoD*. Amino acid exchanges previously identified in σ^32^ [23] are labeled in red.
(EPS)

S3 Fig Spot colony formation assay of the parental DnaKJ depletion strain and its different genetic derivatives under non-depleting conditions. Cells were spotted in different dilutions on PYE xylose and incubated at 37, 30, 22 and 15°C for the number of days indicated.
(EPS)

S4 Fig σ^32^-levels in the parental DnaKJ depletion strain and its different genetic derivatives. The western blot shows σ^32^ steady-state levels after 9 h depletion of DnaKJ in the different genetic backgrounds. Quantifications are based on three independent replicates. Error bars represent standard deviations.
(EPS)

S5 Fig Deletion of *rpoH* restores DNA replication in DnaKJ depleted cells. Phase contrast microscopy and flow cytometry show the cell cycle phenotypes of WT and *ΔrpoH* cells depleted for DnaKJ for 24 h at 30°C.
(EPS)

S6 Fig The *hslVU(Δ201-203HKT)* mutation does not induce the degradation of CtrA, FtsZ or RpoB. The stability of CtrA, FtsZ and RpoB was measured after 8 h of DnaKJ depletion in the *hslU* wild type and the *hslVU(Δ201-203HKT)* background by *in vivo* degradation assays. Quantifications are shown as the means of independent triplicates. Error bars represent standard deviations.
(EPS)

S7 Fig Spot colony formation assays of the DnaKJ depletion strain harboring different *hslU* and *hslV* overexpression constructs under non-depleting and non-inducing conditions. SG400 cells containing either the empty pJS14 or pJS14-based expression constructs for the vanillate-dependent overexpression of *hslV*, *hslU*, *hslU(Δ201-203HKT)*, *hslVU* or *hslVU(Δ201-203HKT)* were spotted on PYE agar allowing for the expression of DnaKJ (+Xyl) and incubated at different temperatures for the time periods indicated.
(EPS)

S8 Fig σ^32^ steady state levels of mutant variants of σ^32^ and *in vivo* stability of the σ^32^(V56A) variant. (A) Steady state levels of σ^32^ in a *P*_*dnaK*_::*Tn* strain harboring either the empty vector pJS14 or a plasmid for the vanillate-inducible overexpression of *rpoH*, *rpoH(V56A)*, *rpoH(V56A; Q202R*) or *rpoH(V56A; YWW*::*AAA)* before and after induction for two hours. (B) *In vivo* stability assay comparing the degradation of wild type σ^32^ and σ^32^(V56A) after one hour of vanillate-dependent overproduction from the high copy plasmid pJS71 in a wild type background. Quantifications are shown as the means of independent triplicates. Error bars represent standard deviations.
(EPS)

S9 Fig Proteomics, additional gene expression comparisons and functional classification of σ^32^-regulated genes. (A) Gene scatter plots for the following comparisons of gene expression and proteomic data: i) 9 h of DnaKJ depletion versus 6 h of DnaKJ depletion, ii) 9 h of DnaKJ depletion in the *rpoH(D252G)* background versus 9 h of DnaKJ depletion in the wild type background, iii) 9 h of DnaKJ depletion in the *rpoH(D252G)* background versus 6 h of DnaKJ depletion in the *rpoH(D252G)* background, iv) protein fold changes after 12 h of DnaKJ depletion versus mRNA fold changes after 6 h of DnaKJ depletion in the *ΔrpoH* background. Red triangles represent proteases and chaperones marked in the heat map in Fig 6C. m, as the slope of the linear regression is shown when the corresponding R^2^ designates a close fit of the data points and allows for a direct comparison of gene induction in samples of the same strain background. (B) Functional classification of genes differentially regulated due to high σ^32^-activity (intersection between the blue and red gene groups (shown in white and pink) in Fig 6B. (C) Volcano plot showing protein abundance fold changes and their corresponding statistical significance. Proteins up- or downregulated 1.5 times with p **≤** 0.05 were considered as differentially regulated.
(EPS)

S10 Fig Proteomap showing changes of the molecular investment into proteins after 12 h of DnaKJ depletion. Alternative visualization of the proteomaps shown in Fig 7A. In this map relative fold changes in the abundance of each protein are shown after 12 h of DnaKJ depletion in comparison to non-depleted cells. The color code indicates the level of up- or downregulation of a protein, where blue corresponds to downregulation and red to upregulation. Statistically insignificant changes are labeled in dark gray. The size of each area in this map is defined by the average (three independent replicates per experimental condition) relative molecular investment (protein relative molecular count multiplied by sequence length).
(EPS)

S1 Table Strains, plasmids and primers used in this study. (XLSX)

S2 Table Mutations identified in the DnaKJ depletion suppressor screen. (XLSX)

S3 Table Processed RNA-seq, ChIP-seq and proteomics data including the hierarchy file used for functionally classifying the *Caulobacter crescentus* NA1000 proteome. (XLSX)

S1 Text Extended materials and methods describing plasmid and strain construction. (DOCX)
