# Supplementary material for: An essential regulatory function of the DnaK chaperone dictates the decision between proliferation and maintenance in Caulobacter crescentus
Source: PLoS Genet. 2017 Dec 27;13(12):e1007148. doi: 10.1371/journal.pgen.1007148 (PMC5760092; doi:10.1371/journal.pgen.1007148)
Supplement: S1 Text — (DOCX) [file pgen.1007148.s014.docx]

**Text S1**

**Extended Materials and Methods**

**Plasmid construction**

**Integrating plasmids**

**Construct for vanillate-dependent expression of *dnaJ* from the chromosomal *vanA* locus (pKJ747):** *dnaJ* was amplified from chromosomal DNA of *C. crescentus* using primers MT1/2. The amplified fragment was cloned into the vector pVVENN-2 using the *Nde*I/*Sac*I restriction sites.

***dnaJ* deletion construct (pKJ814):** To exchange the *kan^R^*-cassette in pNPTS138 against a *spec^R^*-cassette, the latter was amplified from pJS71 using primers MT3/4 and cloned into pNPTS138 using the *Vsp*I/*Xho*I cut sites resulting in pKJ813*.* A fragment encompassing the 605 bp upstream and the first 15 bp of the *dnaJ* encoding sequence was amplified from the *C. crescentus* chromosome using MT7/8. The fragment harboring the 604 bp downstream and the last 30 bp of *dnaJ* was amplified with MT9/10. The *tet^R^*-cassette was amplified from pNPTS-*lon::tet^R^* [[1](#_ENREF_1)] with MT5/6. The fragments were assembled into the *Nhe*I/*Bam*HI precut pKJ813 by Gibson assembly [[2](#_ENREF_2)].

***rpoH* deletion construct (pKJ816):** A fragment containing the 600 bp upstream of the *rpoH* coding sequence was amplified using OFS39/59 and a fragment containing the 612 bp downstream of the gene was amplified with OFS60/42 using chromosomal DNA as a template. The purified fragments were fused in a PCR using OFS39/42 introducing a *Sac*I and *Pst*I cut-site between the homology regions. The obtained fragment was cloned into pNPTS138 using the *Eco*RI and *Hind*III restriction sites resulting in pKJ815. A *tet^R^*-cassette was amplified with OFS53/54 from pNPTS-*lon::tet^R^* [[1](#_ENREF_1)], restricted with *Sac*I/*Pst*I and ligated into the identically digested pKJ815.

***hslU* deletion construct (pKJ918):** The left flanking homology region containing the 695 bp upstream and the first 15 bp of the *hslU* gene was amplified from *C. crescentus* chromosomal DNA using OFS600/602. The right flanking homology region encompassing the last 27 bp and 678 bp downstream of *hslU* was amplified with OFS601/603. The *rif^R^*-cassette was amplified with OKH53/64 using pVMCS-3 [[3](#_ENREF_3)] as template. The fragments were assembled into OFS285/286 amplified pNPTS138 by Gibson assembly.

**Constructs for re-introducing the suppressor mutations (pKJ817 to pKJ823)**: The constructs were generated by assembling PCR-amplified left and right flanking homology regions into *Eco*RI/*Hind*III cut or OFS285/OFS286 amplified (in case of pKJ822 and pKJ823) pNPTS138 via Gibson assembly. Fragments with overlapping sequence stretches including the respective mutations were amplified using the primer pairs: OFS703/718 and OFS719/704 for pKJ817, OFS703/712 and OFS713/704 for pKJ818, OFS703/OFS714 and OFS715/OFS704 for pKJ819, OFS705/716 and OFS706/717 for pKJ820, OFS705/710 and OFS711/706 for pKJ821, OFS634/638 and OFS639/636 for pKJ822 as well as OFS595/598 and OFS597/596 for pKJ823.

**Construct for re-introducing the *rpoBP1272S* mutation (pKJ824):** A DNA stretch containing the 640 bp upstream of *rpoB*, the entire *rpoB* gene containing the missense mutation, the *rpoC* gene as well as 248 bp downstream were introduced into the multiple cloning site of pNPTS138 by Gibson assembly. The insert was amplified as three fragments of similar size using the primer pairs OFS731/734, OFS737/740 and OFS742/745 from genomic DNA of suppressor #24 (Table S3), respectively. The fragments were assembled with the *Eco*RI/*Hind*III-digested pNPTS138 by Gibson assembly. The final construct contained a silent C2922T base pair exchange in *rpoB,* which was not present in the original suppressor.

**Replicating plasmids**

***rpoD* overexpression construct (pKJ825):** *rpoD* was amplified using primers OFS314/315 and cloned into pBVMCS-2 [[3](#_ENREF_3)] using the *Nde*I/*Xba*I restriction sites.

**Empty vector control construct (pKJ826):** Two fragments containing the terminators *rrnB1B2* and *T500* [[4](#_ENREF_4)] in the sequence overlaps were amplified using the primer pairs OFS579/425 and OFS578/583 with pJS71 and pJS14 as template, respectively. The fragments were then joined by Gibson assembly.

**pJS14 based *rpoH*-overexpression constructs (pKJ827 to pKJ830):** The constructs were generated by assembly into OFS441/422 amplified pJS14 in a Gibson assembly reaction. For the amplification of the respective DNA fragment pairs, primer pairs were used as follows: OFS532/510 amplifying a fragment comprising the *rrnB1B2*-terminator fused to the 5’-end of *P_vanR__VanR_P_vanA_* and OFS491/533 amplifying the *rpoH* gene followed by the *T500*-terminator from chromosomal DNA for pKJ827*.* OFS532/178 and OFS177/533 were used for pKJ828 using pKJ827 as a template, OFS532/OFS516 and OFS515/OFS533 for pKJ829 as well as OFS532/OFS417 and OFS418/OFS533 for pKJ830, using pKJ828 as a template*.*

**pJS71 based *rpoH*-overexpression constructs (pKJ912 and pKJ831):** pKJ912 and pKJ831 were generated by amplifying *P_vanR__vanR_P_vanA__rpoH* and *P_vanR__vanR_P_vanA__rpoH(V56A)* from pKJ827 and pKJ828, respectively, using primers OFS237/487 and subsequent ligation into *Sac*I/*Xho*I cut pJS71.

**pJS71 based *rpoH(V56A)*- and *rpoD*-overexpression construct (pKJ881):** A vanillate-dependent *rpoHV56A*- and a xylose-dependent *rpoD*-expression cassette were cloned in opposite directions and separated by the *rrnB1B2* terminator. The fragment containing the *rpoD* gene was amplified using OFS505/506 and the fragment harboring *P_xylX_* flanked by the *rrnB1B2* terminator was amplified with OFS507/508 using pRXMCS-2 [[3](#_ENREF_3)] as a template. *P_vanR__VanR_P_vanA__rpoH(V56A)* was amplified with OFS509/512 using pKJ831 as a template. The fragments were assembled into OFS441/442 amplified pJS71 by Gibson assembly.

**pJS14 based *hslV*- and *hslU*-overexpression constructs (pKJ913-pKJ917):** The constructs were produced by assembling the amplified fragments into *Xho*I/*Sac*I cut pJS14 by Gibson assembly. For the generation of pKJ913, pKJ916 and pKJ917 a *P_vanR__VanR_P_vanA_* fragment was amplified using OFS513/924 for the assembly with either the OFS926/935 amplified *hslV* gene, the OFS926/928 amplified *hslVU* operon or the OFS926/928 amplified *hslVU* operon containing the *hslU(Δ201-203HKT)* mutation (chromosomal DNA of strain KJ841 was used), respectively. In order to construct pKJ914 and pKJ915 a *P_vanR__VanR_P_vanA_* fragment was amplified using OFS513/939 and assembled with OFS928/941 amplified *hslU* or *hslU(Δ201-203HKT)* (chromosomal DNA of strain KJ841 was used), respectively.

**DnaK expression construct pML375-*dnaK*:** Plasmid pML375-*dnaK* was constructed by first creating pENTR-*dnaK* and subsequently recombining it into pML375, a destination vector of pET-His6, using the Gateway LR clonase (Invitrogen). pENTR-*dnaK* was constructed by amplifying *dnaK* using the primers dnaK_F and dnaK_R from the *C. crescentus* chromosome. The fragment was subsequently cloned into pENTR using the pENTR/D-TOPO cloning system (Invitrogen).

**Strain construction**

**Strain allowing for isolated depletion of DnaK (KJ833):** SG400 cells were transformed with pKJ747 to integrate the vector at the *vanA* locus. The strain was verified by PCR.

**DnaJ depletion strain (KJ834):** *C. crescentus* NA1000 was first transformed with pKJ747 to integrate an ectopic copy of *dnaJ* at the *vanA* locus under the control of *P_van_.* The native copy of *dnaJ* was then deleted by transforming the cells with pKJ814, following a two-step recombination procedure [[5](#_ENREF_5)] under ectopic expression of *dnaJ* and selection for tetracycline resistance. Spectinomycin-sensitive clones unable to grow in the absence of vanillate had replaced *dnaJ* by the *tet^R^* cassette and relied on vanillate-dependent expression of *dnaJ* from the *vanA* locus as was also confirmed by colony PCR.

**DnaKJ depletion strains harboring re-introduced suppressor mutations (KJ835 to KJ842):** The suppressor mutations were re-introduced following the two-step recombination procedure after transforming SG400 cells with pKJ817*,* pKJ818*,* pKJ819*,* pKJ820*,* pKJ821*,* pKJ822*,* pKJ823 and pKJ824, respectively. Second recombinants were screened for kanamycin-sensitivity and the capability of growing in the absence of xylose. Positive clones were verified by colony PCR and sequencing. Stocks for cryoconservation were made from cell cultures originating from cells always grown in the presence of xylose to avoid the enrichment of secondary mutations.

***rpoH* knockout strains (KJ843 and KJ844):** The *tet^R^*-marked knockout of *rpoH* was introduced into NA1000 and SG400 cells by two-step recombination selecting for tetracycline resistance after transformation with pKJ816. Colonies grown on the sucrose containing counterselection plates at 30 °C for two days were phenotypically distinguishable by their different growth rates and the fact that only the slower growing colonies were kanamycin-sensitive. Colony PCR as well as Western blotting confirmed that the slower growing cells contained the *rpoH* deletion. We observed that during storage at -80 °C the viability of this strain decreased. For this reason we always re-made the strain prior to performing experiments with it by the procedure described above.

***hslU* knockout strain (KJ900):** The *rif^R^*-marked knockout of *hsU* was introduced into SG400 cells by two-step recombination. Kanamycin-sensitive second recombinants were screened by colony PCR and verified by sequencing.

**Strains harboring replicating plasmids (KJ845 to KJ876, KJ879, KJ901 to KJ911 and KJ919):** Replicating plasmids were transformed by electroporation into the desired strain backgrounds. Strains harboring both a high- and a low-copy plasmid were co-transformed.

**References**

1. Leslie DJ, Heinen C, Schramm FD, Thuring M, Aakre CD, et al. (2015) Nutritional Control of DNA Replication Initiation through the Proteolysis and Regulated Translation of DnaA. PLoS Genet 11: e1005342.

2. Gibson DG, Young L, Chuang RY, Venter JC, Hutchison CA, 3rd, et al. (2009) Enzymatic assembly of DNA molecules up to several hundred kilobases. Nat Methods 6: 343-345.

3. Thanbichler M, Iniesta AA, Shapiro L (2007) A comprehensive set of plasmids for vanillate- and xylose-inducible gene expression in Caulobacter crescentus. Nucleic Acids Res 35: e137.

4. Yarnell WS, Roberts JW (1999) Mechanism of intrinsic transcription termination and antitermination. Science 284: 611-615.

5. Skerker JM, Prasol MS, Perchuk BS, Biondi EG, Laub MT (2005) Two-component signal transduction pathways regulating growth and cell cycle progression in a bacterium: a system-level analysis. PLoS Biol 3: e334.
